# Management of the Venous Anastomoses of a Tertiary Referral Centre in Reconstructive Microvascular Surgery Using Fasciocutaneous Free Flaps in the Head and Neck

**DOI:** 10.3390/jcm14176171

**Published:** 2025-09-01

**Authors:** Nocini Riccardo, Muneretto Carlotta, Lobbia Guido, Zatta Esmeralda, Athena Eliana Arsie, Molteni Gabriele, Arietti Valerio, Barbera Giorgio

**Affiliations:** 1 Unit of Otorhinolaryngology, Head and Neck Department, University of Verona, 37129 Verona, Italy; 2 Maxillo Facial Surgery Unit, Head and Neck Department, Azienda Ospedaliera Universitaria Integrata Verona, 37126 Verona, Italygiorgio.barbera@aovr.veneto.it (B.G.); 3 Department of Otolaryngology, Head and Neck Surgery, IRCCS Azienda Ospedaliero-Universitaria di Bologna, 40138 Bologna, Italy

**Keywords:** head and neck, cancer, reconstruction, radial forearm, venous anastomosis, microsurgery

## Abstract

**Objectives**: The application of fasciocutaneous free flaps for reconstruction of head and neck defects following oncological surgery has increased since the 1970s, coinciding with developments in microvascular techniques. Although reported success rates are between 90% and 99%, flap failure continues to occur, most frequently due to venous congestion. This study examines the rates of re-exploration and flap failure according to the number of venous anastomoses in patients receiving free flap reconstruction after head and neck cancer surgery. **Materials and Methods**: This retrospective analysis included 163 patients who underwent head and neck reconstruction with free flaps (radial forearm free flap [RFFF] and anterolateral thigh flap [ALTF]) at the University Hospital of Verona between January 2019 and June 2024. Variables examined comprised the type of flap performed, donor and recipient vessels utilized, and number of venous anastomoses, as well as the type (end-to-end [ETE] versus end-to-side [ETS]) and site (internal jugular vein versus external jugular vein) of venous anastomosis. **Results**: The overall success rate was 93.3%, with no significant difference between single and dual venous anastomosis groups. Prompt re-exploration upon detecting signs of flap failure is critical, as approximately one-third of such failures may be prevented through timely intervention. **Conclusions**: Single venous end-to-end anastomosis utilizing the internal jugular vein system is typically effective. Further research is warranted to clarify the indications for dual anastomosis involving the external jugular vein system.

## 1. Introduction

Free flaps are commonly used for reconstruction of head and neck defects in major oncological surgery. Since the introduction of free tissue transfer in 1970, pedicled flaps have largely been replaced by free flaps, which facilitate reconstructions aimed at restoring functions such as speech, swallowing, saliva retention, and breathing, in addition to improving esthetic outcomes and overall patient quality of life [1,2,3]. Significant advancements have been achieved due to the enhancement of microvascular techniques and instrumentation since the earliest attempts at reconstruction using free flaps, which were associated with failure rates of 40 to 50%. Currently, success rates range from 90% to 99% [4]. Flap failure remains a possible outcome for surgeons, most often resulting from microvascular complications related to venous congestion instead of arterial occlusion. The occurrence of venous complications is associated with the properties of the venous system, which has lower pressures and thinner walls, making it more susceptible to torsion, kinking, or external compression [5]. Key determinants of thrombosis, a significant contributing factor, include the velocity of blood flow through the anastomosis and endothelial injury, which reveals the subendothelial layer and triggers platelet activation. To reduce the likelihood of thrombotic events, it is critical to maintain venous flow above the threshold level required to prevent thrombogenesis [6].

Efforts to minimize flap failure continue to explore a range of factors that affect flap survival. The optimal number of venous anastomoses remains debated in the literature. Some researchers suggest that adding a second vein might slow blood flow and thereby increase the risk of thrombosis [7]. Some suggest that adding an additional venous anastomosis may maintain adequate outflow if one vein becomes thrombosed, thereby potentially protecting the flap [5,8].

A second venous anastomosis is possible only when two donor veins and two recipient veins are available. The presence of two recipient veins depends on the anatomical location, with certain sites—such as the skull base, hypopharynx, and paranasal sinuses—permitting only a single venous anastomosis due to their configuration or as a result of previous surgical procedures or radiotherapy. The possibility of having two donor veins is determined by the vascular characteristics of the individual flap [9]. A crucial factor in flap success is the meticulous selection of recipient vessels to guarantee adequate vascular supply and minimize the risks of pedicle kinking, tension, or compression. While the cervical region provides access to a diverse array of arteries and veins, it is also influenced by neck movements—including flexion, extension, and rotation—which may impact the outcome of the anastomosis during the immediate postoperative period [10]. Most authors report that the internal jugular vein (IJV) is often considered more reliable than the external jugular vein (EJV) as a recipient vessel [11]. This assessment is based on factors such as its consistent anatomy, high venous flow influenced by negative intrathoracic pressure that may help maintain an open anastomosis site, clear residual microthrombi, and aid in efficient blood drainage from the flap. Additional noted advantages of the IJV include its larger caliber, which can accommodate end-to-side anastomoses, and the flexibility in choosing the anastomosis location to minimize kinking or tension [12]. Conversely, some studies indicate no significant difference in flap survival between the two recipient veins, suggesting that the EJV is also a viable option [10,13,14]. Furthermore, the choice of anastomosis technique—end to end (ETE) versus end to side (ETS)—may impact clinical results. Although many experts regard ETS anastomosis as a secondary option due to its increased technical complexity [15] and some studies link it with higher rates of flap failure [16], it offers notable advantages. Specifically, ETS anastomosis facilitates vascular microsuturing in cases where there is a size mismatch between donor and recipient vessels, thereby minimizing issues related to size discrepancy [10]. Additionally, in situations involving a “vessel-depleted neck”, where at least one internal jugular vein is preserved, ETS anastomosis often emerges as the most practical solution [15]. Patient-related risk factors may contribute to flap complications and failure [17]. It is important to consider patients with a history of prior radiotherapy or surgery in the head and neck region, as anatomical changes such as tissue plane distortion, inflammation, and scarring can occur. These alterations may reduce the availability of suitable recipient vessels and affect the healing process, which can impact the success of the flap [18]. Additionally, patient characteristics and comorbidities—including age, sex, diabetes mellitus, hypertension, vasculopathy, and smoking—may also play a role in flap complications and failure [17]. Additional variables affecting reconstruction outcomes include pedicle length, vascular anastomotic technique, vascular pedicle position, drain placement, and head immobilization; all of these should be assessed on a case-by-case basis.

This study aimed to compare rates of reexploration and flap failure due to venous thrombosis in patients who underwent oncologic surgery for head and neck malignancies, followed by free flap reconstruction using either one or two vein anastomoses. A secondary objective was to evaluate the influence of comorbidity, donor vein site (internal jugular vein versus external jugular vein), and type of anastomosis (end-to-end versus end-to-side) on overall flap success [19,20,21,22].

## 2. Materials and Methods

A retrospective analysis was performed on 163 consecutive patients who underwent microvascular head and neck reconstruction with either a radial forearm free flap (RFFF) or anterolateral thigh flap (ALTF) for the treatment of malignant tumors in the head and neck region. The study included only patients who received a free flap transfer during the same oncologic resection procedure. Patients with trauma or benign pathologies were not included in the analysis. The procedures were performed in the Department of Otolaryngology and Oral and Maxillofacial Surgery between January 2019 and June 2024. Eligible cases were identified through a review of operative logs documenting free flap surgeries at the Head and Neck Department, University Hospital of Verona (Italy).

Demographic data, comorbidities, and individual risk factors were collected for each patient, including age, gender, medical history (tumor type and site, previous surgery or radiotherapy in the head and neck region), and history of hypertension, diabetes, vasculopathy, and smoking. Patient charts were examined to record the type of free flap performed, donor and recipient vessels used, number of venous anastomoses, as well as the type (end-to-end [ETE] vs. end-to-side [ETS]) and site (internal jugular vein vs. external jugular vein) of venous anastomosis.

Whenever feasible, surgeries involved a two-team approach: one team responsible for flap harvesting and another for tumor resection and neck dissection. For all flaps, arterial anastomosis was initially completed using an end-to-end technique with a size-matched vessel from the external carotid system in the cervical area. Subsequently, venous anastomosis was performed either in an end-to-side or end-to-end manner. The ETE anastomosis was generally used, while ETS was applied when a significant vessel mismatch existed or suitable venous vessels were unavailable. The internal jugular vein, or more commonly one of its branches such as the thyrolinguofacial trunk, was chosen as the recipient vessel when appropriate. The external jugular vein was considered only if alternative options were not available. Flap choice was determined by the defect type and characteristics of the available donor sites. A secondary venous anastomosis was performed if venous return in the initial anastomosis was insufficient, as indicated by an inconclusive milking test or vein tension. In these situations, the second anastomosis was typically created on another venous branch, most often the external jugular vein. Microvascular anastomosis involved handsewn sutures with Prolene 9/0 under microscopic magnification. During flap harvesting and pedicle dissection, lidocaine and papaverine were applied to the pedicle to reduce the risk of vasospasm. One to two minutes before detaching the pedicle flap, an intravenous bolus of heparin was administered. After raising the flap, heparinized solution and urokinase were flushed into the pedicle vessels. All surgical procedures were carried out under general anesthesia, and patients were monitored postoperatively in the intensive care unit using invasive mechanical ventilation and analgosedation until at least the following morning. Thromboprophylaxis therapy was provided daily during the postoperative period until patient discharge, and antibiotic therapy was routinely maintained for 14 days. Antithrombin III blood levels were checked during the first three postoperative days, and corrective measures were taken when levels fell below 70%. Attention was given to the patient’s head positioning to avoid stretching or kinking the anastomosis. The standard protocol includes elevating the backrest of the bed to 45 degrees and maintaining head alignment with the torso using a dedicated pillow for at least the first three postoperative days. After surgery, patients were kept intubated and sedated overnight in the intensive care unit, with vital signs monitored and maintained within defined parameters. Mean arterial pressure (MAP) was maintained between 70 and 90 mmHg without the use of inotropes or vasopressors. On the first postoperative day, patients were moved to the ward to begin rehabilitation. Mobilization was gradually initiated once considered safe, typically on day 2 or 3, while observing precautions to prevent stretching or compressing the vascular pedicle. All flaps underwent postoperative monitoring at regular intervals—every 4 h during the first 3 days, followed by every 6 h for the subsequent 3 days—using clinical assessments of flap skin paddle temperature and color, turgor, capillary refill time, and dermal bleeding response to needle stick. The patency of arterial and venous pedicles was evaluated with a Doppler pen during each clinical examination. Any signs indicative of flap compromise were promptly documented, leading to immediate surgical exploration of the anastomosis in the operating room. Outcome measures included flap survival rate after an average follow-up period of six months, frequency of re-exploration procedures, and salvage rate following surgical revision. Flap failure was defined as complete removal of the flap due to necrosis. Intraoperative findings at the time of re-exploration and any additional complications were also recorded. All observational data were systematically entered into Microsoft Excel and subsequently analyzed by the Department of Statistics at the University of Verona.

## 3. Results

A total of 163 flaps were performed: 82 anterolateral thigh (ALT) and 81 radial forearm free flaps (RFFF), used to reconstruct defects from oncological surgery. Tumor site and histotype distributions are detailed in Table 1 and Table 2.

Flap success rates were 93.3% overall, 91.5% for ALT, and 93.8% for RFFF. Of 11 failures, 7 were arterial and 4 venous. Reintervention was required in 6.7% of cases; 3 of 4 flaps revised for venous issues were salvaged, and 1 required a pedicle flap. Only 1 ischemic flap was saved with re-exploration; the rest were replaced by pedicle or secondary free flaps. The salvage rate was 36.4%, with an overall success rate, including salvaged flaps, of 95.7% (Table 3).

Of the 151 flaps, a single venous anastomosis was performed, while 12 flaps underwent a second venous anastomosis (Table 4). In the group with two venous anastomoses, only one flap required surgical revision due to arterial failure and was successfully salvaged. In contrast, within the single-vein group, 10 flaps exhibited clinical signs of vascular compromise: 6 due to arterial thrombosis or compression, and 4 due to venous insufficiency. None of the six flaps experiencing arterial failure could be salvaged; consequently, three pedicle flaps and three free flaps were performed as alternatives. Of the four flaps affected by venous insufficiency, three were salvaged through prompt reexploration in the operating room, whereas one was unsuccessful and subsequently replaced with a pedicle flap.

In 150 flap procedures, the internal jugular vein or one of its branches was selected as the recipient vein, while the external jugular vein was used in 7 cases. Among patients who underwent dual venous anastomosis, both the internal and external jugular veins were utilized in 6 out of 12 cases. The overall flap success rate in the internal giugular vein (IJV) group was 95.3%. A total of 10 flaps failed in this group: 4 due to venous insufficiency—of which 3 were salvaged after surgical revision and 1 with a pedicle flap—and 6 due to arterial complications, subsequently replaced by 3 pedicle flaps and 3 free flaps. In the external jugular vein (EJV) group, no flap failures were recorded; however, among the 6 flaps in the EJV + IJV group, 1 required revision due to ischemia and was successfully salvaged (Table 5).

With respect to the types of anastomosis, 130 end-to-end (ETE) and 33 end-to-side (ETS) procedures were performed. Within the two-vein group, 8 patients underwent dual ETE anastomoses, while in 4 cases both ETS and ETE techniques were utilized (specifically, tyrolinguofacial trunk plus IJV in three cases, and EJV in the remaining case). No significant difference in flap survival was observed between ETS and ETE methods. The impact of anastomosis type on flap success rates is detailed in Table 6.

Table 7 shows the links between comorbidities, age, tobacco use, preoperative radiation, prior surgery, and flap survival.

The overall rate of other complications was 8.6%. Specifically, we observed 8 cases of flap dehiscence, 4 instances of partial loss of flap tissue, 1 occurrence of a submental oro-cutaneous fistula, and 1 case of dehiscence at the donor site surgical wound.

## 4. Discussion

Flap success relies on effective arterial and venous anastomosis, with venous failure being the leading cause of complications. To address this, many surgeons add a second venous anastomosis. Two meta-analyses have shown that dual venous anastomoses reduce thrombosis and flap failure rates compared to a single anastomosis [21,22].

Evan et al., in their meta-analysis, demonstrated that one and two venous anastomoses yield similar outcomes regarding flap loss rate, venous congestion, and thrombosis in ALT flaps. Notably, the double venous anastomosis group experienced a significantly reduced flap take-back rate, albeit with a substantial increase in operative time [23]. In our clinical experience, the decision to perform a second venous anastomosis is made intraoperatively based on the donor vein’s diameter and the flap’s size. If the surgeon determines that venous drainage may be insufficient, two donor veins are harvested for the flap. For neck procedures, at least two recipient veins are routinely prepared to anticipate primary anastomosis failure or the need for revision. The data demonstrate that the utilization of a single vein generally ensures effective drainage, notwithstanding the fact that all revisions were observed within the single-vein group. In three such cases, kinking or external compression of the vein was identified during revision, suggesting that a second venous anastomosis might have mitigated the drainage inadequacy. Similarly, in the sole instance of intraluminal thrombosis, an additional vein would likely have provided alternative drainage. However, adding a second vein increased the average operative time by 45 min and introduced further considerations regarding the risk of kinking and stretching of the peduncle. While no complications were observed in the two-vein group—though this group comprised fewer cases—we advise against routinely implementing a second vein solely to preempt a theoretically low complication rate. Future research should focus on refining selection criteria for patients who may genuinely benefit from a second venous anastomosis, thus avoiding unnecessary prolongation of complex procedures, especially in patients with multiple comorbidities. Some studies have examined various techniques to reduce operative time and support surgeons in maintaining proper ergonomic posture. For example, technological devices like the VITOM^®^ 3D system have been used; however, additional research is required [24].

According to our data, Lin et al. recommend utilizing a single vein due to an absence of significant differences in most reported outcomes [25]. Similarly, Tawa et al. have indicated that a single venous anastomosis with the internal jugular vein may be sufficient to maintain adequate venous flow [10]. Most prior studies identify the internal jugular vein (IJV) and its branches as optimal recipient vessels for free tissue transfer [26,27]. Notably, although the incidence of IJV thrombosis following functional neck dissection ranges from 14 to 33%, microvascular flaps anastomosed to the IJV system continue to demonstrate higher success rates [28,29]. Based on these findings, our practice predominantly selects the internal jugular vein system as the recipient vessel over the superficial venous system. The external jugular vein is reserved for select cases, such as those involving radical neck dissection (RND) or previously irradiated necks where vascular availability is compromised and the internal jugular vein system is unsuitable for anastomosis. This approach is consistent with existing literature, including a meta-analysis by Yin et al. [12], which reported a lower incidence of thrombosis and flap failure following venous anastomosis in the internal jugular vein (IJV) system compared to the external jugular vein (EJV) system. Notably, our experience indicates that utilizing the superficial venous system does not adversely affect flap viability. In our series, all seven anastomoses performed using the external jugular vein were successful, with no cases of failure observed. Consequently, we consider the external jugular vein a viable alternative, particularly in situations where the IJV has been sacrificed during radical or modified neck dissection, or when the EJV is the only suitable recipient vessel. This statement should be considered only indicative since the number of cases is extremely limited; therefore, studies with larger cohorts are definitely necessary, even though the results appear promising. It should be noted that the EJV may be at increased risk for thrombosis due to its lower flow, smaller caliber, and susceptibility to intimal damage during surgical manipulation [30].

When a second venous anastomosis is required, we prefer to perform one anastomosis on the superficial venous system and another on the deep system. This approach addresses concerns regarding system failure from factors such as thrombosis, compression, or other complications. By utilizing two macrosystems—the internal and external jugular veins—each can provide support should the other become compromised. In our limited series of six patients, no instances of flap failure were observed; however, further research is required to validate these findings.

The choice of the most appropriate venous drainage for radial forearm flap remains controversial. The flap is provided by a double venous drainage: the deep comitants veins, with a maximum diameter of 2 mm, and the superficial subcutaneous veins that drain into the cephalic vein, with a diameter from 3 to 6 mm. The selection of the most reliable venous drainage system for anastomosis remains a significant challenge. The majority of the evidence indicates that the superficial system should be preferred because the bigger diameter of the vessel facilitates anastomosis. Other autors favored the use of the comitants veins (VC), arguing that the deep system is responsible for the majority of the venous drainage. Flow studies demonstrated that VCs with an adequate caliber constituted the dominant drainage system [26,31,32,33]. In our practice we preferred to use a single anastomosis with a comitant vein in order to reduce operating time, without encountering difficulties in performing the anastomosis due to smaller vessel size.

Several studies have demonstrated no statistically significant difference in venous patency between end-to-end (ETE) and end-to-side (ETS) anastomoses in size-matched vessels. However, ETS anastomosis has been associated with a lower rate of venous thrombosis in cases of size-discrepant veins [15,34,35]. Based on our clinical experience, we preferentially perform ETE anastomosis when feasible and deemed appropriate by the surgeon. The rationale for preferring the end-to-end technique for anastomoses stems from our belief that the end-to-end approach allows for greater ease in placing sutures, resulting in fewer technical errors and a cleaner execution of the suture line. Additionally, we believe it carries a lower risk of vessel kinking. Nevertheless, we remain prepared to utilize ETS anastomosis when indicated. This technique is typically selected when no suitable veins are available, except for the internal jugular vein. Additionally, we consider employing ETS anastomosis when the vessel caliber mismatch exceeds one-third, which we regard as the maximum correctable discrepancy using surgical methods [36]. Finally, ETS anastomosis is preferred if there are concerns about potential kinking with ETE anastomosis.

Each case is carefully assessed, taking into account the length, caliber, and anatomical relationship of the recipient and donor vessels; the final decision regarding the type of anastomosis is made to optimally balance these factors.

Among the 33 ETS procedures performed, nine were necessitated by nonviable internal jugular vein branches. In nine additional cases, venous size mismatch prompted the use of ETS anastomosis to minimize vessel caliber discrepancy. The remaining fifteen cases involved ETS anastomosis based on the surgeon’s judgment regarding pedicle geometry, length, or position. In our series, selecting ETS anastomosis when ETE was unsuitable for the reasons mentioned above did not appear to adversely affect flap success rates.

Multiple studies indicate that advanced age is not considered a contraindication for performing free flaps in the head and neck regions. These findings include patients aged over 60, following the World Health Organisation (WHO) definition of ‘elderly’ [37]. In this population, the mean age at surgery was 66. Age was analyzed as a risk factor by dividing the cohort into those above and below 80 years old. No significant difference in flap failure rates was observed between these age groups, suggesting that age alone does not impact overall flap success unless accompanied by severe comorbidities.

Squamous cell carcinoma represented the most common histotype in this cohort (147 patients), consistent with existing literature. Although this type represents a substantial proportion of cases annually, national awareness of oral cavity squamous cell carcinoma remains limited [38,39].

The selection of reconstruction technique generally depends on the size and characteristics of the defect resulting from cancer ablation. Another important consideration in selecting a free flap type is its reliability. This study found comparable survival rates for RF and ALT flaps. However, specific criteria or indications to guide the choice between RF and ALT flaps have not been established. For oral cavity tumors involving T3 or T4 tongue and requiring extended glossectomy, some literature, such as Gazzini et al. [40], suggests a preference for ALT over RF. In cases of palate defects, the radial forearm flap, particularly when used with the technique described by Nocini R. et al. [41], is often selected. Both the ALT and radial forearm flap are commonly utilized in double flap cases for soft tissue reconstruction in the head and neck region, as reported by Mannelli G. et al. [42].

A total of 110 patients (80%) presented with comorbidities, although the reported success rate remained favorable. The success rate for free flaps in this study was consistent with previously published data; however, six cases required revision surgery due to microvascular complications. Literature indicates that venous thrombosis, which is primarily associated with kinking or twisting of the vein (mechanical stress) and intraoperative hypotension, occurs at a higher rate than arterial thrombosis. Arterial thrombosis leads to flap failure via ischemia, occurring less frequently but with a lower salvage rate. This difference may be due to the fact that detecting flap congestion is generally easier than identifying ischemic flaps, as venous congestion is often accompanied by edema and hematoma formation. In contrast to these reports, more cases of arterial spasm than venous thrombosis were observed in this study [14,43,44].

## 5. Conclusions

Although our study did not yield statistically significant data, the findings support the validity of our protocol for minimizing venous complications in free fasciocutaneous tissue transfer procedures of the head and neck. Our findings suggest that a single vein anastomosis, ideally utilizing the internal jugular vein and an end-to-end approach, demonstrates high efficacy along with a low rate of complications. As the sample size in this study was limited, further investigations involving larger cohorts are necessary to evaluate the effectiveness of the external jugular vein system, determine its association with low complication rates as a stand-alone anastomosis, and explore the potential benefits of implementing dual venous anastomoses that incorporate both internal and external venous systems.

This study is subject to several limitations, including its retrospective design, an uneven distribution of cases that precluded meaningful statistical analysis, and its single-center setting. Future research should focus on larger, prospective, multicenter studies to further address these issues and improve clinical outcomes in free tissue transfer.

## Figures and Tables

**Table 1:** Tumor site distribution.

Tumor Site	
Oral cavity	128 (78.5%)
Oropharynx	20 (12.3%)
Larynx	3 (1.8%)
Parotid gland	4 (2.5%)
Skin	2 (1.2%)
Others *	6 (3.7%)

* includes 3 ipopharynx, 2 orbit, 1 maxillary sinus.

**Table 2:** Hystotipe distribution * includes myoepithelioma and sarcomatoid carcinoma.

Histotype	
Squamous cell carcinoma	147 (78.5%)
Adenoid cystic carcinoma	6 (3.7%)
Others *	10 (6.1%)

**Table 3:** Flap success/reexploration.

	TOTAL	ALT	RFFF
Total flaps	163	82	81
Reexploration rate	6.7%	7.3%	6.2%
Venous issues at reexploration	4	2	2
Successful venous reexploration	3 (75%)	2 (100%)	1 (50%)
Arterial issues at reexploration	7	4	3
Successful arterial reexploration	1 (14.3%)	1 (25%)	0 (0%)
Overall flap success	95.7%	96.4%	95.1%

**Table 4:** The effect of 1 vs. 2 veins on flap success.

	One-Vein Group	Two-Vein Group
Total flaps	151	12
Reexploration rate	6.6%	8.3%
Venous issues at reexploration	4	0
Successful venous reexploration	3	-
Arterial issues at reexploration	6	1
Successful arterial reexploration	0	1
Overall flap success rate	95.4%	100%

**Table 5:** The effect of recipient venous system on flap success IJV = internal giugular vein EJV = external giugular vein.

	IJV	EJV	IJV + EJV
Total flaps	150	7	6
Reexploration rate	6.6%	0	16.6%
Venous issues at reexploration	4	0	0
Successful venous reexploration	3 (75%)	-	-
Arterial issues at reexploration	6	0	1
Successful arterial reexploration	0	-	1
Overall flap success rate	95.3%	100%	100%

**Table 6:** The effect of anastomosis type on flap success.

	ETE	ETS
Total flaps	130	33
Reexploration rate	6.9%	6%
Venous issues at re-exploration	3	1
Successful venous re-exploration	2 (66.6%)	1 (100%)
Arterial issues at re-exploration	6	1
Successful arterial reexploration	1 (16.6%)	0
Overall flap success rate	95.4%	96.9%

**Table 7:** The effect of age, sex and comorbidities on flap succes.

	Total No. Cases	Survived	Failed
Age < 80	142 (87.1%)	132	10 (7%)
Age > 80	21 (12.9%)	20	1 (4%)
Male	104 (63.8%)	97	7 (6.7%)
Female	59 (36.2%)	55	4 (6.8%)
Diabetes	18 (11.1%)	18	0 (0%)
Hypertension	95 (58.3%)	90	5 (5.2%)
Vasculopathy	64 (39.2%)	62	2 (3.1%)
Tobacco use	85 (52.1%)	77	8 (9.4%)
Previous radiotherapy	20 (12.4%)	18	2 (10%)
Previous surgery	34 (21.2%)	31	3 (8.8%)

## Data Availability

All the data used in this article is presented within the article itself.
